# New Laplace and Helmholtz solvers

**DOI:** 10.1073/pnas.1904139116

**Published:** 2019-05-06

**Authors:** Abinand Gopal, Lloyd N. Trefethen

**Affiliations:** ^a^Mathematical Institute, University of Oxford, Oxford OX2 6GG, United Kingdom

**Keywords:** Laplace equation, Helmholtz equation, rational functions, scattering

## Abstract

Numerical algorithms based on rational functions are introduced that solve the Laplace and Helmholtz equations on 2D domains with corners quickly and accurately, despite the corner singularities.

The Laplace and Helmholtz equations are the basic partial differential equations (PDEs) of potential theory and acoustics, respectively. Suppose a domain Ω bounded by a polygon P is given and (to begin with the Laplace case) we seek the unique function u(x,y) that satisfies $\Delta u=\partial^{2}u/\partial x^{2}+\partial^{2}u/\partial y^{2}=0$ in Ω and matches a given function h at the boundary. More generally, P might have curved sides meeting at corners, and the boundary data might involve derivatives as well as function values. It is convenient to represent the coordinates by a complex variable z=x+iy, so we write the boundary condition as u(z)=h(z) for z∈P.

The standard techniques for solving such a problem numerically are the finite element method (FEM) (1) and boundary integral equations (2). However, these methods face a challenge in calculating accurate solutions because of singularities at the corners (3). The mathematical basis of an algorithm for meeting this challenge, whose computational implications have not been noticed before, is a theorem in the field of approximation theory published by D. J. Newman in 1964 (4). Newman considered the approximation of f(x)=|x| on the interval [−1,1] by a rational function, that is, a quotient of polynomials r(z)=p(z)/q(z). He showed that whereas polynomial approximations converge at best at the very slow rate $\|f-p_{n}\|=O\left(n^{-1}\right)$, rational approximations can achieve much faster “root-exponential” convergence $\|f-r_{n}\|=O\left(\exp\left(-C\sqrt{n}\right)\right)$ with C>0. Here n is the degree of a polynomial or rational function, which is defined in the latter case as the maximum of the degrees of p and q.

Our algorithm achieves root-exponential convergence for solving PDEs by approximating u(z) by the real part of a rational function, u(z)=Rer(z). Any such approximation is a harmonic function in Ω, that is, a solution of the Laplace equation, provided r has no poles in Ω. Finding rational approximations to given data is a difficult nonlinear problem in general (5). Here, however, we know that the singularities of u lie at the vertices of P. This suggests the idea, motivated by Newman’s result and related computational experience, of prescribing poles of r outside Ω a priori in a configuration with exponential clustering near each vertex. Specifically, our rational functions take the form

$$r\left(z\right)=\overset{N_{1}}{\underset{j=1}{\sum }}\frac{a_{j}}{z-z_{j}}+\overset{N_{2}}{\underset{j=0}{\sum }}b_{j}{\left(z-z_{*}\right)}^{j},$$ [1]

where $z_{*}$ and $\left\{z_{j}\right\}$ are fixed points interior and exterior to Ω, respectively, and $\left\{a_{j}\right\}$ and $\left\{b_{j}\right\}$ are complex unknowns ($N=2N_{1}+2N_{2}+1$ real degrees of freedom in total, since $b_{0}$ can be taken to be real). The second series reflects the fact, going back to Runge in 1885, that polynomials are effective at approximating smooth functions in the plane. The first series is targeted at the corner singularities, and one might expect that its poles $z_{j}$ would have to be delicately chosen to be effective. However, in a mathematical result to be published elsewhere, we have proved that a straightforward realization of this idea, relying on no unknown parameters, is enough to guarantee the existence of approximations [**1**] with root-exponential convergence. To find such functions computationally, one exploits the fact that since the poles are prescribed the problem is linear. Expansion coefficients are found by least-squares fitting in sample points on the boundary, a routine problem of linear algebra involving a matrix of dimensions about 3N×N. The sample points need not be chosen carefully, so long as they are clustered exponentially near the corners like the poles. In typical problems on polygons with up to eight vertices, N≈1,000 suffices to give accuracy to 8 or 10 digits, complete with an a posteriori accuracy guarantee derived from the maximum principle. Both speed and accuracy, which is currently limited by ill-conditioning of the matrices, can be improved by good choices of pole placement parameters, a subject for future research.

Our method can be regarded as a variant of the method of fundamental solutions (MFS), in which solutions are also approximated via finite sums (6). The MFS differs from our approach in that each term is normally a monopole or point charge rather than a dipole (an exception in the context of Maxwell equations is ref. 7), it is not normally applied with exponential clustering (an exception is ref. 8), and it is not founded upon results of rational approximation theory (although rational functions are used in ref. 5).

Fig. 1 shows a computed solution in an L-shaped domain. For a problem that is generic in the sense of having singularities at the corners, the simplest choice of boundary data is $u\left(z\right)=h\left(z\right)={\left[\text{Re}z\right]}^{2}=x^{2}$. Our code approximates u successively with N=42, 82,138,…,1,002 degrees of freedom, at which point 10-digit accuracy is achieved. The figure also shows the accuracy as a function of $\sqrt{N}$, revealing a straight line corresponding to root-exponential convergence. For a wide range of problems, this performance is representative. Typically we find coefficients in <1 s on a laptop running MATLAB in 16-digit floating-point arithmetic, and then each evaluation of u(z), with guaranteed accuracy all the way to the corners, takes a few tens of microseconds.

**Fig. 1.**
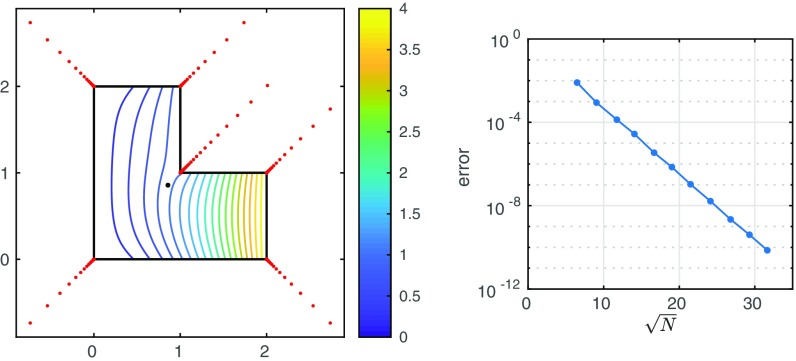
Laplace equation in an L-shaped domain. Poles are clustered exponentially near vertices and a least-squares problem is solved on the boundary to find coefficients for a global representation [**1**] of the solution accurate to 10 digits. The black dot in the interior marks the point $z_{*}$ of [**1**]. The curve of maximal error on the boundary shows root-exponential convergence.

The numerical solution of PDEs has been at the heart of scientific computing since computers were invented, and the Laplace equation in a planar domain is as fundamental a problem in this area as any. There are two main classes of methods for solving such problems: FEM and boundary integral equations. To gather information on how our approach compares with these, in November 2018 we posed the problem of Fig. 1 as a challenge to the international numerical analysis community via the email list NA Digest (9). Specifically, we asked for a computation of u(0.99,0.99) to eight digits of accuracy (the exact value is 1.02679192610…). This led to responses from about 20 experts around the world. About half the responses recommended FEM algorithms and software such as FEniCS, Firedrake, IFISS, and PLTMG. In this approach, the set of functions on Ω is approximated by a finite-dimensional linear space and one solves a matrix problem to find a good candidate in that space. The FEM is noted for its flexibility, enabling problems much more complicated than ours to be solved effectively, in three as well as two dimensions. However, although hp-adaptive FEM can also achieve root-exponential convergence near singularities (1), this requires advanced implementations, and whereas all respondents were able to calculate a solution to two to four digits of accuracy, only two came close to eight digits. For example, one researcher used 158,997 fifth-order triangular elements near the reentrant corner and achieved six correct digits. Our assessment is that although it is possible to solve the Laplace equation on a domain with corners to high accuracy by the FEM, this involves a significant computation requiring a high level of expertise and tools.

The other well-known methods are boundary integral equations, which are advantageous because of good conditioning and because the solution is represented in one dimension, along the boundary, rather than two dimensions in the domain (2). Here one begins by solving an integral equation to determine a density function ρ(z), such as the equation $h\left(z\right)=-\pi \rho \left(z\right)+\int_{P}K\left(z,\zeta \right)\rho \left(\zeta \right)|d\zeta |$, where ∠(z−ζ,ν(ζ)) is the angle between z−ζ and the inward normal to P at ζ and K(z,ζ)=cos(∠(z−ζ,ν(ζ)))/|z−ζ|. This is a “double layer potential” formulation, whose solution ρ(z) can be interpreted as a distribution of dipole charge density along the boundary. Once ρ is found, the solution to the Laplace problem is evaluated at a point z by computing the integral $u\left(z\right)=\int_{P}K\left(z,\zeta \right)\rho \left(\zeta \right)|d\zeta |$. In general ρ will be smooth along the sides and singular at the corners, introducing challenges in evaluating the integrals. Additionally, the kernel K is singular, so that even away from the corners an accurate evaluation near the boundary may be a nontrivial task. However, experts have developed powerful techniques for such quadratures, and five respondents to our inquiry produced results accurate to the specified eight digits and sometimes much more. It seems clear that if one wants an accurate solution to Laplace problems in corners, integral equations are the most powerful of the existing technologies. However, there is not much software available, and the solutions communicated to us were produced by experts running their own codes.

Whereas integral equations make use of a continuous distribution of dipoles ρ(z) on the boundary, the method described here can be interpreted as using a finite sum of dipoles (delta functions) beyond the boundary. This has the advantage that evaluation of u(z) takes exactly the same form [**1**] regardless of z, requiring no special calculations when z is near a boundary arc or a corner. One may regard this as a zero-dimensional representation of the solution, whose complexity is determined only by the complexity of the function being represented, not by that of Ω or P.

Our method may be generalized to other elliptic PDEs, notably the Helmholtz equation $\Delta u+k^{2}u=0$, which models time-harmonic propagation of acoustic or electromagnetic waves at frequency k. For a Helmholtz problem in a domain exterior to a scattering body with scattered fields satisfying the Sommerfeld radiation condition, we modify [**1**] to

$$\begin{array}{ll} & \overset{N_{1}}{\underset{j=1}{\sum }}H_{1}\left(k|w_{j}|\right)\left(a_{j}\text{Re}\frac{w_{j}}{\left|w_{j}\right|}+b_{j}\text{Im}\frac{w_{j}}{\left|w_{j}\right|}\right)\\ & +\overset{N_{2}}{\underset{j=0}{\sum }}H_{j}\left(k|w_{*}|\right)\left(c_{j}\text{Re}\frac{w_{*}^{j}}{\left|w_{*}^{j}\right|}+d_{j}\text{Im}\frac{w_{*}^{j}}{\left|w_{*}^{j}\right|}\right), \end{array}$$ [2]

where $w_{j}=z-z_{j}$, $w_{*}=z-z_{*}$, and $H_{j}$ are Hankel functions of the first kind. (Our target is problems with small or medium values of k; large k brings new challenges requiring other methods.) We cluster singularities exponentially near corners in the interior of the scatterer, and $z_{*}$ lies in the interior too. The least-squares problem is now complex, with complex coefficients $\left\{a_{j}\right\},\ldots ,\left\{d_{j}\right\}$ chosen to cancel a signal incident at the boundary; the “sound-soft” case has zero total field at the boundary and the “sound-hard” case has zero normal derivative. Fig. 2 shows two sound-soft example problems solved to four-digit accuracy. In each case the solution took about 2 s on a laptop, with about 250 μs for each point evaluation afterward. Again the convergence is root-exponential. (Rigorous a posteriori estimates will now require a modification of the maximum principle.)

**Fig. 2.**
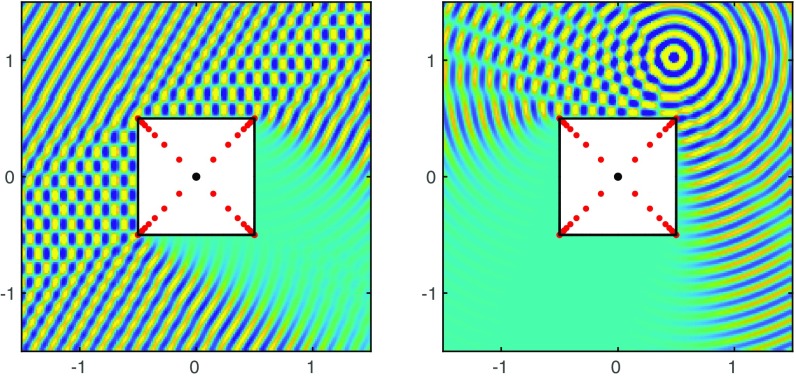
Helmholtz equation. Solutions to $\Delta u+k^{2}u=0$ with k=50 in the exterior of a square (the real part is plotted). The incident signal on the left is a plane wave oriented at 30°, and on the right, a point oscillation $H_{0}\left(k|z-z_{0}|\right)$ with $z_{0}=1/2+i$.

There is a historical context that may shed light on the paucity of previous literature on solving PDEs via rational functions and their generalizations. The mathematicians of the 19th century concentrated on functions that were analytic or piecewise analytic, that is, smooth and representable by convergent Taylor series. Most physical applications are of this kind. In the 20th century, however, mathematicians turned to new challenges of less-smooth functions, developing advanced tools for analyzing fine distinctions of regularity (i.e., smoothness). Overwhelmingly, such tools became the standard framework for computational mathematics, too, and in particular FEM experts almost invariably derive and analyze their algorithms in the language of Sobolev spaces (1), in which precise distinctions are made, for example, between a function with one derivative of smoothness and a function with one and a half derivatives. This kind of analysis entails a bias toward low-accuracy methods tuned to problems with limited smoothness. The simplicity and speed of the methods proposed here are a reminder that there is also a place for numerical analysis based on less pessimistic smoothness assumptions.

In closing, let us summarize some pros and cons of the method. It is limited to problems of small or medium size in two dimensions (at least as currently developed) and to certain constant-coefficient PDEs with known special solutions. It faces challenges of ill-conditioning that are not yet understood and do not arise with boundary integral equations, and since the convergence is only root-exponential, it would not be competitive at very high accuracies (say, >15 digits). However, it is extremely simple and flexible, it requires no analysis of singularities or derivation of integral equations or quadrature formulas, it is not limited to convex or straight-sided domains, it delivers a global (hence perfectly smooth) solution as an explicit formula with an a posteriori error bound, and for medium-accuracy solutions (say, 4 to 10 digits) it can be extraordinarily fast. We believe this approach holds great promise and that developing it is an exciting challenge for the years ahead.
